# Public mental health problems during COVID-19 pandemic: a large-scale meta-analysis of the evidence

**DOI:** 10.1038/s41398-021-01501-9

**Published:** 2021-07-09

**Authors:** Xuerong Liu, Mengyin Zhu, Rong Zhang, Jingxuan Zhang, Chenyan Zhang, Peiwei Liu, Zhengzhi Feng, Zhiyi Chen

**Affiliations:** 1 Department of Medical Psychology, Army Medical University, Chongqing, China; 2 School of Psychology, Southwest University, Chongqing, China; 3 Cognitive Psychology Unit, The Institute of Psychology, Faculty of Social and Behavioural Sciences, Leiden University, Leiden, Netherlands; 4 Department of Psychology, University of Florida, Gainesville, FL USA

**Keywords:** Scientific community, Psychiatric disorders

## Abstract

The coronavirus disease 2019 (COVID-19) pandemic has exposed humans to the highest physical and mental risks. Thus, it is becoming a priority to probe the mental health problems experienced during the pandemic in different populations. We performed a meta-analysis to clarify the prevalence of postpandemic mental health problems. Seventy-one published papers (*n* = 146,139) from China, the United States, Japan, India, and Turkey were eligible to be included in the data pool. These papers reported results for Chinese, Japanese, Italian, American, Turkish, Indian, Spanish, Greek, and Singaporean populations. The results demonstrated a total prevalence of anxiety symptoms of 32.60% (95% confidence interval (CI): 29.10–36.30) during the COVID-19 pandemic. For depression, a prevalence of 27.60% (95% CI: 24.00–31.60) was found. Further, insomnia was found to have a prevalence of 30.30% (95% CI: 24.60–36.60). Of the total study population, 16.70% (95% CI: 8.90–29.20) experienced post-traumatic stress disorder (PTSD) symptoms during the COVID-19 pandemic. Subgroup analysis revealed the highest prevalence of anxiety (63.90%) and depression (55.40%) in confirmed and suspected patients compared with other cohorts. Notably, the prevalence of each symptom in other countries was higher than that in China. Finally, the prevalence of each mental problem differed depending on the measurement tools used. In conclusion, this study revealed the prevalence of mental problems during the COVID-19 pandemic by using a fairly large-scale sample and further clarified that the heterogeneous results for these mental health problems may be due to the nonstandardized use of psychometric tools.

## Introduction

Since the end of 2019, the coronavirus disease 2019 (COVID-19) outbreak has continued to spread worldwide. Researchers rapidly identified the cause of COVID-19 to be the transmission of serious acute respiratory syndrome by a novel coronavirus (SARS-CoV-2) [1]. Unfortunately, due to the lack of effective cures and vaccines, the ability of public medical systems to guard against COVID-19 is deteriorating rapidly. Although approved vaccines are now available, their safety is still a concern [2, 3]. Further, because of reports regarding the potential to be reinfected with COVID-19, public panic is still spreading even though COVID-19 transmission has been contained substantially [4]. To date, projections regarding the end of the COVID-19 pandemic around the world are still far from optimistic. There were more than 158.95 million confirmed cases and 3.30 million deaths by May 11, 2021 (supported by Johns Hopkins University), a situation that has led to unprecedented losses and stress.

COVID-19 not only threatens physical health but has also led to mental health sequelae (i.e., loss of family, job loss, social constraints and uncertainty, and fear about the future) [5–7]. In general, mental health problems, including depression and anxiety, have had major negative impacts on the public during the COVID-19 pandemic [8, 9]. Previous studies showed that mental health problems, such as depression, anxiety, insomnia, and post-traumatic stress disorder (PTSD), suddenly increased after the COVID-19 outbreak: 53.8% of respondents rated the psychological impact of the outbreak as moderate or severe; 16.5% of participants reported moderate to severe depressive symptoms; 28.8% of participants reported moderate to severe anxiety symptoms; and 24.5% of participants showed psychological stress [10]. Moreover, such mental health problems were worse in confirmed patients and healthcare workers. As a typical example, one early study revealed acute anxiety symptoms in 98.84% of confirmed patients and depression symptoms in 79.07% of confirmed cases [11]. In addition, an early investigation concerning the mental health status of 400 public health workers found that 31% of public health workers had anxiety symptoms, and 24.5% of them had depressive symptoms [12]. In this vein, it seems that the mental health sequelae of the COVID-19 pandemic warrant more attention. In addition, with the development of the epidemic situation, long-term isolation due to the increasing number of confirmed and suspected patients has caused losses to life and property, which has not only caused considerable psychological stress in the population but has also had physiological effects, such as insomnia and PTSD.

In brief, the COVID-19 pandemic has exposed public health to dramatic risks and resulted in unacceptable mental and physiological stresses. Despite considerable research, two critical concerns regarding mental health problems during the COVID-19 pandemic remain. One concern in previous studies is that the conclusions regarding the prevalence of these mental health problems are highly heterogeneous, irrespective of whether they are derived from original investigations or meta-analyses [13, 14]. Another is that early investigations were almost all done during the peak of the COVID-19 pandemic and thus may overestimate the scale of mental health problems. Thus, the main purpose of this study is to provide comprehensive statistical results regarding the impact of COVID-19 on individual mental health through a large-scale meta-analysis of the existing research in this field and to provide an evidence-based reference for the prevention and control of psychological crises during this pandemic. It is noteworthy that this study employs a larger data pool than any of the existing meta-analyses to date. Further, much effort has been made to perform an in-depth investigation of the patterns of mental health problems triggered by the COVID-19 pandemic, including population-, region-, and measurement-specific patterns.

## Materials and methods

To improve reproducibility and standardization, all the pipelines and protocols were in line with the Cochrane Handbook and were double-checked by using the PRISMA checklist [15]. This meta-analysis has been preregistered on OSF for open access (10.17605/OSF.IO/A5VMK).

### Search strategy and selection criteria

**Fig. 1:**
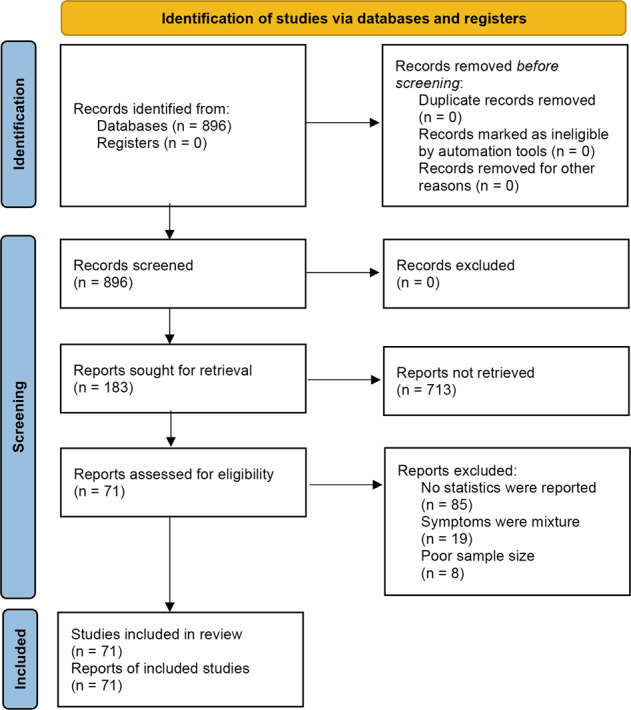
Flow chart of the study selection process in the 2020 PRISMA protocol. This flowchart is coincide with the broad-certified 2020 PRISMAstatement. Small sample size was predefined as < 30 participants.

A systematic search was conducted for studies published from January 1, 2020 to July 1, 2020 (the period from the commencement of the outbreak to its initial control in China) in PubMed, EMBASE, the Cochrane Library, EBSCO, Web of Science, CNKI (Chinese database), WANGFANG DATA, the Chinese Biomedical Literature Service System, and public information release platforms (WeChat Subscription or microblogs). According to the indices of the various databases, keywords, including “2019 novel coronavirus,” “COVID-19,” “novel coronavirus pneumonia,” “NPC,” “2019-nCoV,” “mental health,” “anxiety,” “depression,” “psychological health,” “sleep,” “insomnia,” “Posttraumatic stress disorder,” and “PTSD,” were adopted to retrieve published surveys of psychological status during the COVID-19 epidemic from January 1, 2020 to July 1, 2020. In addition to identifying any target studies that may have been missed, we checked the reference list of each selected paper. The population was divided into three categories according to the probable psychological stress intensity experienced: public health workers, confirmed patients, and the general population (see Fig. 1, Supplemental information, and Table S1).

### Data extraction and quality assessment

The following data were extracted from each article by two researchers independently: study type; total number of participants; participation rate; region; percentage of physicians, nurses, and other healthcare workers screened in the survey; number of male and female participants; assessment methods used and their cutoffs; and the total number and percentage of participants who screened positive for depression, anxiety, insomnia or PTSD. If any of this information was not reported, the necessary calculations (e.g., transforming the percentage of healthcare workers to the number of healthcare workers) were performed. The accuracy of the extracted or calculated data was confirmed by comparing the collection forms of the two investigators.

In addition, two authors independently evaluated the risk of bias of the included cross-sectional studies using a modified form of the Newcastle-Ottawa scale. Potential disagreements were resolved by a third author. Specifically, the quality assessment criteria were as follows: sample representativeness and size; comparability between respondents and nonrespondents; ascertainment of depression, anxiety, and insomnia; and adequacy of the descriptive statistics. The total quality scores ranged between 0 and 5; studies scoring ≥3 points were regarded as having a low risk of bias, while studies scoring <3 points were regarded as having a high risk of bias (see Table S1).

### Encoding and statistical analysis

The two investigators (XL and MZ), who performed the literature search, also extracted the data from the included studies independently. Disagreements were resolved with the third investigator (ZC) or by consensus. Then, the following variables were extracted: author, date of publication, age, gender, region, sample size, method, number of positive cases, and positivity rate. All these analytical procedures were performed with the CMA software (V3). In particular, given the heterogeneity within and between studies, random-effects models were used to estimate the average effect and its precision, which would give a more conservative estimate of the 95% confidence intervals (CIs). The *I*^2^ statistic and Cochran’s *Q* test were conducted to assess statistical heterogeneity.

Prior researchers held that the fixed-effects model is ideally suited to the meta-analysis of a nonheterogeneous data pool (*I*^2^ < 50%, *P* value ≥0.1) [16]. Conversely, the random-effects model should be used when there is heterogeneity between the studies (*I*^2^ > 50%). According to the factors that may affect the heterogeneity between studies, moderation analysis was further carried out for distinct cohorts (i.e., health workers, confirmed and suspected patients, the general population) and distinct sample sources (China, other countries). A funnel chart was created for visual inspection to determine whether the included studies showed publication bias; Egger’s test and Kendall’s test for the quantitative analysis of publication bias were also used, with *p* > 0.05 indicating no publication bias.

## Results

In the current study, 896 Chinese and English studies were initially retrieved. According to the inclusion and exclusion criteria, 71 papers were eligible for inclusion in the data pool for the meta-analysis, and the total number of respondents reached 146,139 (see Table 1 and Table S2).

**Table 1:** Summary of characteristics of included studies.

Author	Publication time	Data collection time	Region	Groups	Sample size	Age	Gender		Anxiety			Depression			Insomnia			PTSD		
							Male	Female	Positive	Rate	Scale	Positive	Rate	Scale	Positive	Rate	Scale	Positive	Rate	Scale
Luo Q [23]	2020.4.17	2020.2.10–20	CN	HW	171	18–41+	41	130	77	45%	GAD-7	68	39.80%	PHQ-9	NA	NA	NA	69	40.40%	PSS-10
Yang L [24]	2020.4	2020.2	CN	GP	521	19–60+	142	379	6	1.20%	PQEEPH	48	9.20%	PQEEPH	NA	NA	NA	NA	NA	NA
Zhu X [25]	2020.5.8	2020.1.30–2.13	CN	GP	6395	NA	2176	4219	2812	43.97%	GAD-7	3413	53.37%	PHQ-9	NA	NA	NA	2071	32.38%	SRQ-20
Zhu K [26]	2020.4.30	2020.2.28–3.5	CN	GP	1264	NA	707	557	234	18.51%	SCARED	NA	NA	NA	NA	NA	NA	NA	NA	NA
Yin J [27]	2020.4	2020.2.3–2.5	CN	HW	1266	NA	320	946	333	26.30%	Questionnaire on mental health of medical staff	348	27.50%	Questionnaire on mental health of medical staff	NA	NA	NA	NA	NA	NA
Wang C [28]	2020.4.22	NA	CN	GP	896	17–30	254	642	102	11.38%	SCL-90	217	24.22%	SCL-90	105	11.72%	SCL-90	NA	NA	NA
HuangPu M [29]	2020.4.3	NA	CN	HW	326	28.62 ± 12.23	0	326	266	81.60%	SCL-90	229	70.25%	SCL-90	NA	NA	NA	NA	NA	NA
Cao J [30]	2020.3.20	2020.2	CN	CP	148	18–79	70	78	32	21.63%	SAS	74	50.00%	SDS	NA	NA	NA	NA	NA	NA
Liu X [31]	2020.3.27	2020.2.1–18	CN	HW	1097	18–0	19	1078	302	27.53%	GAD-7	408	37.19%	PHQ-9	234	21.33%	ISI	111	10.12%	SRQ-20
Song W [32]	2020.3	2020.1.28–2.20	CN	GP	1078	∼60	553	525	445	41. 28%	SCL-90	358	33. 21%	SCL-90	NA	NA	NA	NA	NA	NA
Xu Y [33]	2020.4.15	2020.2.7–2.15	CN	HW	360	23–56	69	291	68	18.90%	SAS	138	38.40%	SDS	NA	NA	NA	NA	NA	NA
Yuan J [34]	2020.4.2	2020.1.27–2.24	CN	CP	145	45.2 ± 16.6	64	81	126	86.90%	SAS	102	70.3%	SDS	NA	NA	NA	NA	NA	NA
Wei L [35]	2020.4.21	NA	CN	HW	2150	NA	873	1277	924	43.00%	GAD-7	946	44.00%	PHQ-9	774	36.00%	PSQI	NA	NA	NA
Zhong Y [36]	2020.3	2020.2.11–2.12	CN	HW	136	23–55	19	117	83	61. 02%	GAD-7	NA	NA	NA	NA	NA	NA	NA	NA	NA
Xu Y [37]	2020.4.16	NA	CN	HW	42	NA	NA	NA	37	88.10%	SAS	NA	NA	NA	NA	NA	NA	NA	NA	NA
Zhang Y [38]	2020.3.11	2020.1.29–2.6	CN	HW	224	NA	56	168	67	29.90%	SAS	NA	NA	NA	NA	NA	NA	NA	NA	NA
Liu H [39]	2020.2.28	2020.2	CN	HW	427	20–50+	93	334	96	22.48%	GAD-7	84	19.67%	PHQ-9	NA	NA	NA	NA	NA	NA
Song M [40]	2020.4.21	2020.1.28–2.4	CN	GP	3111	0–60+	NA	NA	1210	38.89%	GAD-7	NA	NA	NA	NA	NA	NA	NA	NA	NA
Li X [41]	2020.4	2020.2.18–2.21	CN	HW	213	∼30–60	6	207	30	14.00%	NA	42	19.70%	NA	NA	NA	NA	NA	NA	NA
Li S [42]	2020.4	2020.2.6–2.8	CN	GP	396	12.82 ± 2.61	199	197	87	22.00%	SCARED	NA	NA	NA	NA	NA	NA	NA	NA	NA
Zhang X [43]	2020.3.12	2020.1–2	CN	HW	133	20–50	13	120	78	58.65%	SCL-90	56	42.11%	SCL-90	NA	NA	NA	NA	NA	NA
Cheng J [44]	2020.3.4	2020.2.1–2.2	CN	CP	59	NA	24	35	28	47.46%	Self-made questionnaire	NA	NA	NA	NA	NA	NA	NA	NA	NA
Cao X [45]	2020.4.28	NA	CN	CP	65	18–74	NA	NA	17	26.10%	SCL-90	13	20%	SCL-90	NA	NA	NA	NA	NA	NA
Zhang J [11]	2020.4.28	NA	CN	CP	86	60–92	39	47	85	98.84%	SAS	68	79.07%	SDS	NA	NA	NA	NA	NA	NA
Fan Y [46]	2020.4	2020.2.23–2.25	CN	GP	4148	20.59 ± 1.52	1121	3027	2082	50.20%	SAS	NA	NA	NA	NA	NA	NA	NA	NA	NA
Fang X [47]	2020.2	2020.2.1–2.6	CN	GP	51	23–77	25	26	12	23.53%	GAD-7	16	31.37%	PHQ-9	NA	NA	NA	NA	NA	NA
Chen Y [48]	2020.3	2020.2.3–2.16	CN	HW	711	NA	315	396	154	21.66%	GAD-7	185	26.02%	PHQ-9	NA	NA	NA	NA	NA	NA
Chen J [49]	2020.2.22	NA	CN	HW	206	NA	63	143	23	11.17%	SAS	NA	NA	NA	NA	NA	NA	NA	NA	NA
Chang J [50]	NA	2020.1.31–2.3	CN	GP	3881	18+	1434	2447	1032	26.60%	GAD-7	821	21.16%	PHQ-9	NA	NA	NA	NA	NA	NA
Yang Y [51]	2020.3.13	NA	CN	GP	176	NA	58	118	71	40.30%	SAS	NA	NA	NA	NA	NA	NA	NA	NA	NA
Wang M [52]	2020.4.28	2020.2.1–2.18	CN	GP	1501	50–65	576	925	444	29.58%	GAD-7	612	40.77%	PHQ-9	432	28.78%	ISI	337	24.50%	SRQ-20
Fang P [53]	2020.2.29	2020.1.24–2.1	CN	GP	36,636	NA	13,848	22,788	NA	NA	NA	NA	NA	NA	8903	24.30%	Self-made questionnaire	NA	NA	NA
Li C [54]	2020.4	2020.2.8–2.11	CN	HW	205	NA	30	175	NA	NA	NA	NA	NA	NA	NA	NA	NA	104	50.73%	PCL-C
Mei J [55]	2020.3	2020.1.15–2.10	CN	CP	70	36.24 ± 9.20	15	55	NA	NA	NA	NA	NA	NA	6	8.57%	PSQI	NA	NA	NA
Huo M [56]	2020.2.26	NA	CN	GP	930	NA	434	496	253	27.20%	GAD-7	251	26.98%	PHQ-9	NA	NA	NA	NA	NA	NA
Lei [57]	2020.3.26	2020.2.4–2.10	CN	GP	1593	18–39	617	976	132	8.30%	SAS	233	14.60%	SDS	NA	NA	NA	NA	NA	NA
Ahmed [58]	2020.4.1	NA	CN	GP	1074	14–68	571	503	311	29.00%	BAI	397	37.10%	BDI	NA	NA	NA	NA	NA	NA
Lai [17]	2020.3.23	2020.1.29–2.3	CN	HW	1257	NA	293	964	560	44.60%	GAD-7	634	50.40%	PHQ-9	427	34%	ISI	899	71.50%	IES-R
Wang C [10]	2020.3.3	2020.1.31–2.2	CN	GP	1210	12–59	396	814	348	28.80%	DASS-21	200	16.50%	DASS-21	NA	NA	NA	98	8.10%	IES-R
Huang Y [59]	2020.4.6	2020.2.3–2.17	CN	GP	7236	NA	7236	3284	3952	35.10%	GAD-7	1454	20.10%	CES-D	1317	18.20%	PSQI	NA	NA	NA
VIDYADHARA [60]	2020.5.12	2020.4.23–4.30	CN	GP	500	NA	174	326	158	31.50%	DASS-21	130	26.00%	DASS-21	NA	NA	NA	NA	NA	NA
Tang W [61]	2020.5.8	2020.2.20/27	CN	GP	2501	16–27	1525	960	NA	NA	NA	224	9.00%	PHQ-9	201	8.08%	Sleep durations	67	2.70%	PCL-C
Wang S [62]	2020.5.17	2020.1.30–2.7	CN	HW	123	20–50+	22	111	25	20.31%	SAS	9	7.00%	SDS	47	38%	PSQI	NA	NA	NA
Casagrande [63]	2020.5.7	2020.3.18–4.2	CN	GP	2291	18–29	580	1708	735	32.10%	GAD-7	NA	NA	NA	1308	57.10%	PSQI	174	7.60%	PCL-5
Cao W [64]	2020.3.19	NA	CN	GP	7143	NA	2168	4975	1779	24.00%	GAD-7	NA	NA	NA	NA	NA	NA	NA	NA	NA
Wu J [65]	2020.2	NA	CN	HW	106	24–39	21	85	84	79.25%	SAS	NA	NA	NA	68	64.15%	PSQI	NA	NA	NA
Xu M [66]	2020.2	NA	CN	HW	41	26–35	4	37	16	39.02%	SCL-90	2	4.88%	SCL-90	NA	NA	NA	NA	NA	NA
Kuang Z [67]	2020.3	2020.2.8	CN	GP	422	18–50+	NA	NA	NA	NA	NA	NA	NA	NA	NA	NA	NA	NA	NA	NA
Wang Y [68]	2020.3	2020.2.6–2.8	CN	GP	396	8–18	199	197	NA	NA	NA	41	10.40%	DSRS	NA	NA	NA	NA	NA	NA
Cai H [69]	2020.2	2020.1.31–2.4	CN	GP	22,302	NA	7398	14,904	NA	NA	NA	7226	32.40%	PHQ-9	NA	NA	NA	NA	NA	NA
Tian Y [70]	2020.2	NA	CN	GP	87	32.18 ± 6.89	47	40	NA	NA	NA	NA	NA	NA	NA	NA	NA	2	2. 3%	Post-traumatic stress disorder self-rating scale
Huang X [71]	2020.2.16	NA	CN	HW	50	38.28 ± 11.9	18	32	11	22.00%	SAS	NA	NA	NA	NA	NA	NA	NA	NA	NA
Cai F [72]	2020.2.23	NA	CN	HW	48	28–39	11	37	42	87.50%	SCL-90	2	4.17%	SCL-90	NA	NA	NA	NA	NA	NA
Qi J [12]	2020.2	NA	CN	HW	400	21–51+	105	295	124	31.00%	SAS	98	24.50%	SDS	NA	NA	NA	NA	NA	NA
Andrea A [73]	NA	2020.3.15–4.15	Italy	GP	131	NA	68	63	NA	NA	NA	30	22.90%	(PHQ-9)	NA	NA	NA	NA	NA	NA
Selçuk Özdin [74]	NA	2020.4.14–4.16	Turkish	GP	343	NA	174	169	155	45.10%	HAI	81	23.60%	HADS	NA	NA	NA	NA	NA	NA
Deblina R [75]	NA	2020.3.2–3.24	Indian	GP	662	NA	323	339	387	58.50%	Self-made questionnaire	NA	NA	NA	NA	NA	NA	NA	NA	NA
Jessica [76]	NA	2020.3.25–4.6	Spain	GP	93	NA	33	60	22	24.00%	Telephone-based survey	27	29%	Telephone-based survey	NA	NA	NA	NA	NA	NA
Chrysi K [77]	2020.5.17	2020.4.4–4.9	Greece	GP	1000	NA	680	320	425	42.50%	STAI	743	74.30%	CES-D	430	43.00%	RASS	NA	NA	NA
Rosen [78]	NA	2020.4.15/4.17	US	GP	303	18–95	206	97	209	69.00%	BAI	NA	NA	NA	127	41.90%	SUDS	NA	NA	NA
Du J [79]	2020.5.31	2020.2.13–2.17	CN	HW	134	NA	53	81	28	20.10%	BAI	17	12.70%	BDI-II	NA	NA	NA	NA	NA	NA
Guo J [80]	NA	NA	CN	HW	11,118	NA	2802	8316	1940	17.45%	SAS	3497	31.45%	SDS	NA	NA	NA	NA	NA	NA
Huang J [81]	NA	NA	CN	HW	230	NA	43	187	53	23.04%	SAS	NA	NA	NA	NA	NA	NA	NA	NA	NA
Liu C [82]	NA	NA	CN	HW	512	NA	79	433	64	12.50%	SAS	NA	NA	NA	NA	NA	NA	NA	NA	NA
Liu Z [83]	NA	NA	CN	HW	4679	NA	828	3852	753	16.10%	SAS	1619	34.60%	SDS	NA	NA	NA	NA	NA	NA
Lu W [84]	2020.3.21	2020.2.25–2.26	CN	HW	2299	NA	514	1785	569	24.70%	HAMA	268	11.70%	HAHD	NA	NA	NA	NA	NA	NA
Qi J [85]	2020.5.13	NA	CN	HW	1306	NA	256	1050	NA	NA	NA	NA	NA	NA	594	45.50%	AISPSQI	NA	NA	NA
Zhang C [86]	2020.3.27	2020.1.29–2.3	CN	HW	1563	NA	270	1293	698	44.70%	GAD-7	792	50.70%	PHQ-9	564	36.10%	ISI	NA	NA	NA
Zhang W [87]	2020.3.30	NA	CN	HW	2182	NA	781	1401	228	10.40%	GAD-2	232	10.60%	PHQ-2	739	33.90%	ISI	NA	NA	NA
Zhu Z [88]	NA	NA	CN	HW	5062	NA	759	4303	1218	24.06%	GAD-7	681	13.45%	PHQ-9	NA	NA	NA	NA	NA	NA
Tan B [89]	2020.4.6	2020.2.19–3.13	Singapore	HW	470	NA	149	321	68	14.50%	DASS-21	42	8.90%	DASS-21	NA	NA	NA	36	7.70%	IES-R

All studies are cross-sectional. The absolute number of patients for each category is included within the parentheses.

*W* healthcare workers, *GP* general people, *CP* confirmed patients, *CH* China, *US* United States, *NA* not available.

### Heterogeneity test

The results of the heterogeneity test on the prevalence of mental problems in patients with COVID-19 showed that the heterogeneity across studies was large (*I*^2^ > 98%, *P* < 0.05), which suggested that the random-effects model was needed to analyze the total effect. Importantly, to increase the robustness of the results and reduce the heterogeneity between studies, population, nationality, and subgroup were analyzed as possible moderators.

### Prevalence of mental problems

**Fig. 2:**
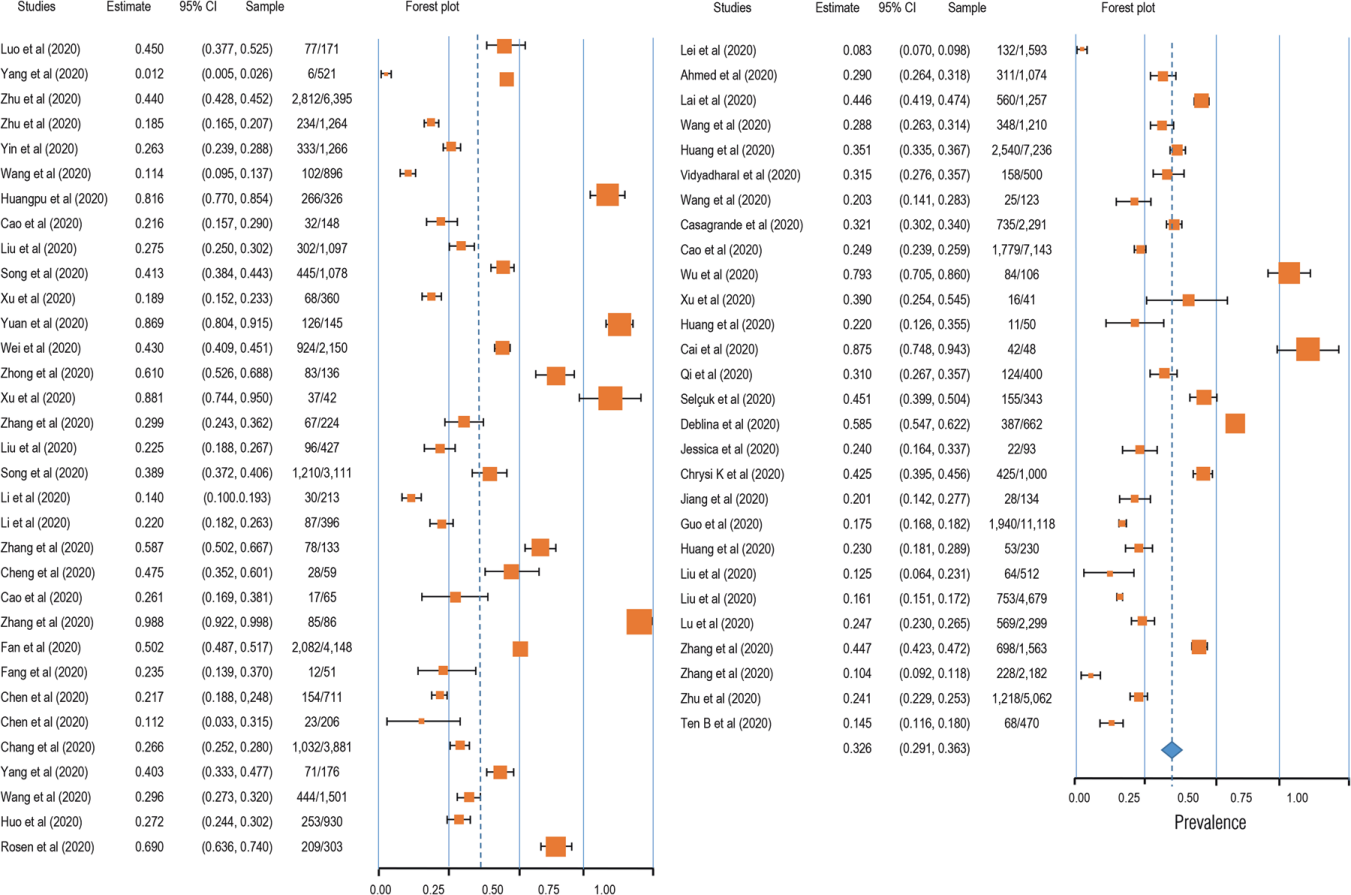
Forest plot for meta-analytic results of the prevalence of anxiety symptoms. The squares colored by orange represent the point estimation foreffect towards corresponding study, with the large square size for high effect size. The orange diamond represent meta-analytic effect size.

**Fig. 3:**
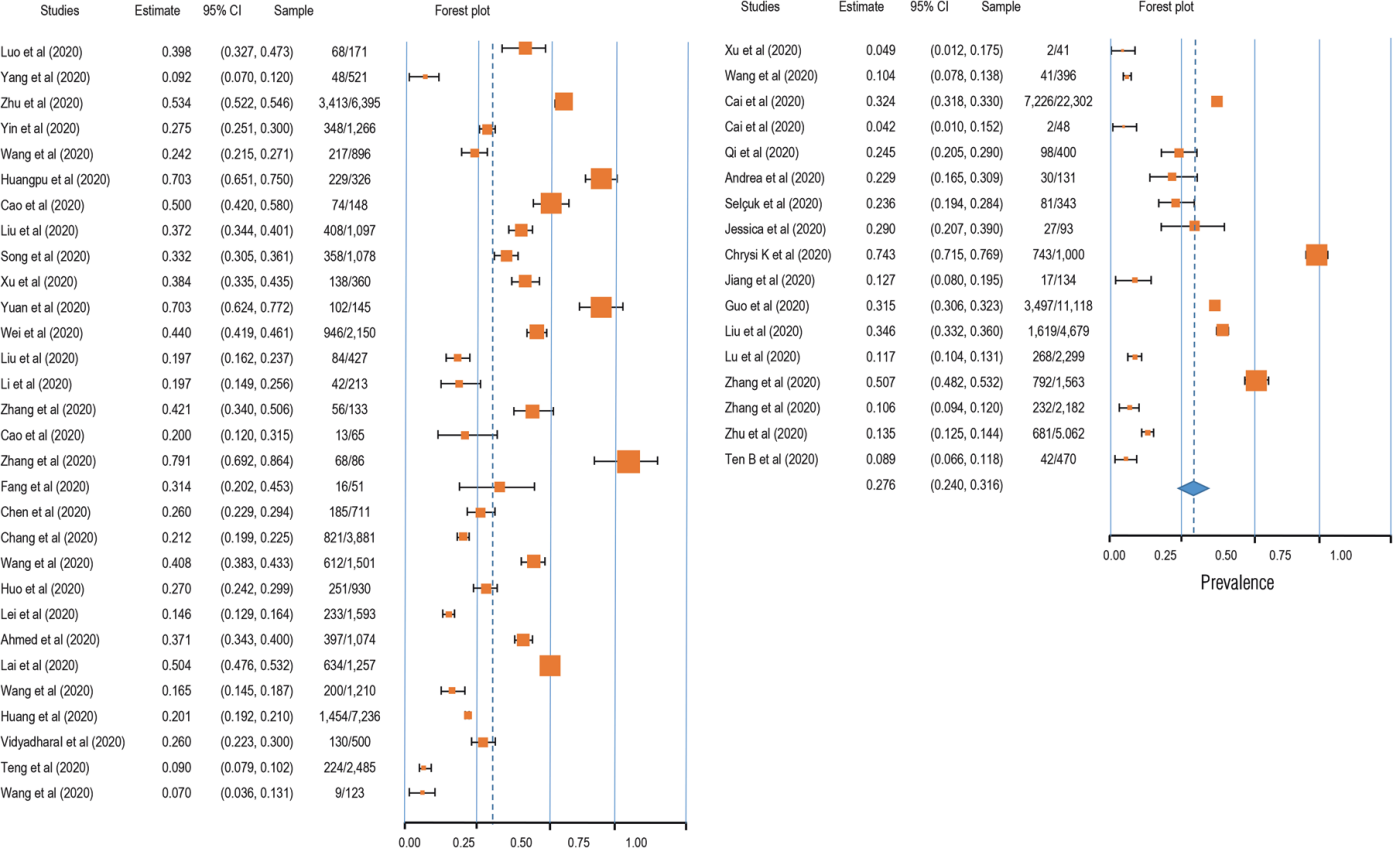
Forest plot for meta-analytic results of the prevalence of depression symptoms. The squares colored by orange represent the point estimation foreffect towards corresponding study, with the large square size for high effect size. The orange diamond represent meta-analytic effect size.

**Fig. 4:**
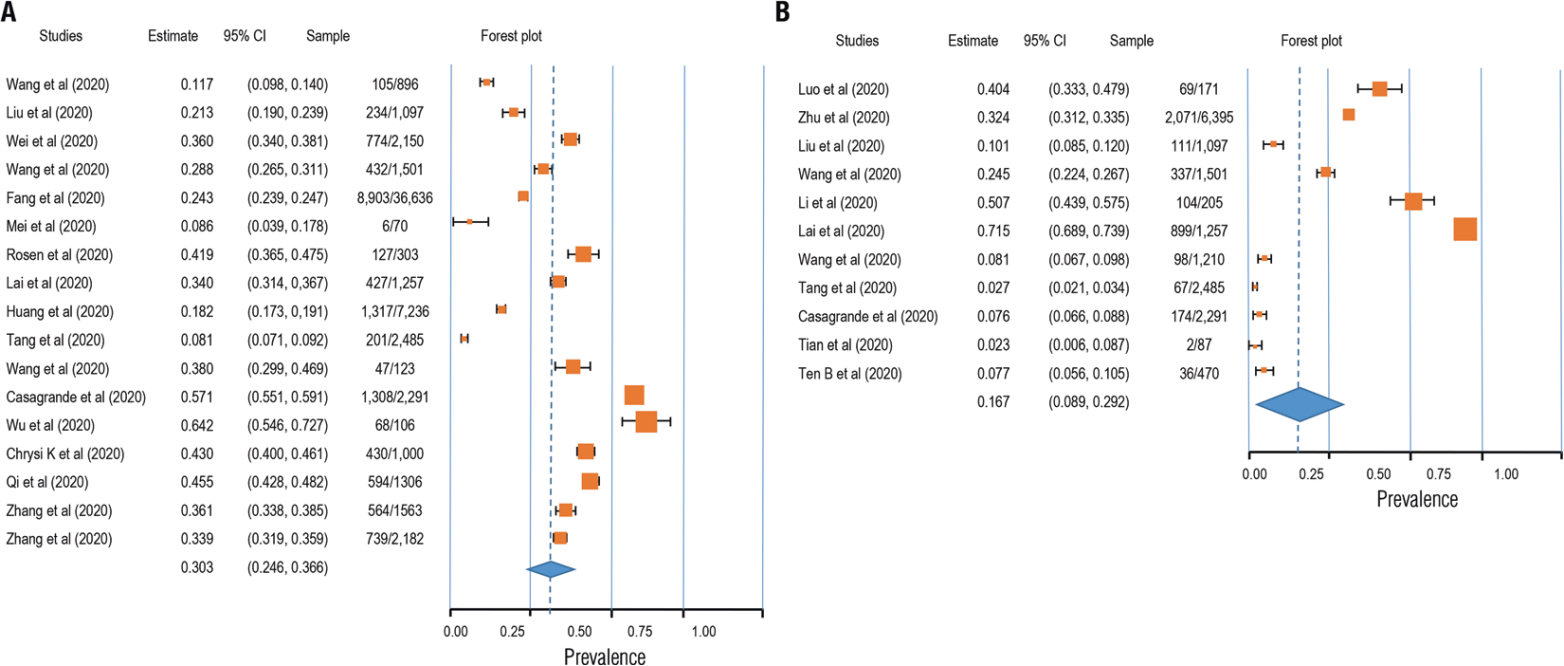
Forest plot for meta-analytic results of the prevalence of insomnia and PTSD symptoms. The squares colored by orange represent the point estimation for effect towards corresponding study, with the large square size for high effect size. The orange diamond represent meta-analytic effect size.

Four symptoms related to stress were selected as the mental problems, and the related symptoms and symptom groups were analyzed according to the definitions given in each study. The prevalence of anxiety was 32.6% (95% CI: 29.1–36.3; *N* = 86,035, see Fig. 2). In addition, the prevalence of depression was 27.60% (95% CI: 24.0–31.6; *N* = 90,156, see Fig. 3). Likewise, insomnia prevalence during the COVID-19 pandemic was 30.30% (95% CI: 24.6–36.6; *N* = 62,202, see Fig. 4A). Finally, 16.70% of participants were found to meet the criteria for PTSD during the COVID-19 pandemic in this meta-analysis (95% CI: 8.9–29.2; *N* = 17,169, see Fig. 4B).

### Moderation analysis

Given the high heterogeneity, we assumed that there were some potential moderators, including the cohort (confirmed patients, healthcare workers, and the general population), region (China and other countries), and measurement tool. The results demonstrated a significantly higher prevalence of mental health problems in confirmed patients than in others (see Table S3). Further, the prevalence of mental health problems was found to be lower in China than in other countries. In addition, these findings derived from the moderation analysis revealed the moderating role of the measurement tool, with the results varying significantly across different scales (see Table S3 and Figs. S1–3).

### Publication bias assessment

A funnel plot was first used for qualitative analysis of the publication bias. As shown in Figure S4, a symmetrical distribution was found for the four psychological symptoms. In addition, Begg’s rank test was performed to quantitatively analyze the publication bias. The results showed that there was no publication bias in the studies regarding anxiety (Kendall’s tau = 0.044, *p* = 0.614), depression (Kendall’s tau = −0.046, *p* = 0.647), insomnia (Kendall’s tau = −0.096, *p* = 0.592), or PTSD (Kendall’s tau = −0.145, *p* = 0.533).

## Discussion

In this study, a meta-analysis was performed to clarify the mental health situation in the population during the COVID-19 pandemic with respect to anxiety, depression, sleep problems, and PTSD. The results showed that the detection rate of anxiety symptoms in a total of 86,035 cases was 32.6% (95% CI: 29.1–363); the detection rate of depression symptoms in a total of 90,156 cases was 27.6% (95% CI: 24.0–31.6); the detection rate of insomnia symptoms in a total of 62,202 cases was 30.3% (95% CI: 24.6–36.6); and the detection rate of PTSD symptoms was 16.7% in a total of 17,169 cases (95% CI: 8.9–29.2). Furthermore, the moderator analysis showed that mental health problems (i.e., anxiety and depression) had the highest prevalence in COVID-19 patients, and fewer anxiety, depression, and sleep problems were observed in healthcare workers than in the general population. Overall, this study provided solid evidence of the mental health situation during the COVID-19 pandemic and indicated the potential heterogeneity across cohorts, regions, and measurement tools.

Furthermore, regarding anxiety symptoms, health workers accounted for 32.7% (95% CI: 27.9–38.2) of the detection rate; the general population accounted for 29.5% (95% CI: 25.2–34.3). A total of 25.8% (95% CI: 20.4–31.0), and 25.3% (95% CI: 20.4–32.0) of depressive symptoms were found in health workers and the general population, respectively. The highest detection rate of insomnia, which was 37.3% (95% CI: 32.1–42.8%), was found in health workers, and the general population represented 26.1% of cases (95% CI: 18.2–36.1). The detection rate of PTSD was 30.6% (95% CI: 9.1–65.9) in health workers and just 9.3% (95% CI: 4–19.8) in the general population. Moving beyond previous studies, this meta-analysis covered the latest COVID-19-related articles and examined more publications than its predecessors. In contrast to the existing research conclusions, this study found that the mental health problems of healthcare workers are the same as those of the general population, suggesting that the existing research may overestimate the mental health problems of healthcare workers (i.e., one study showed that 50.4% of healthcare workers reported symptoms of depression, 44.6% symptoms of anxiety, and 34.0% insomnia) [17]. This may be because in the early stage of COVID-19, the pressures experienced by healthcare workers were considerable due to the sudden workload and lack of adequate understanding of the COVID-19 pandemic. However, in later stages, as an understanding of COVID-19 improved, healthcare workers became familiar with the situation and gained a more comprehensive understanding of the disease. This led to higher self-regulation ability under the circumstance of the epidemic even though the stress level of the first-line workers was high. Therefore, a very important conclusion of this study is that the mental health problems of healthcare workers are not as serious as previously thought, and lagging research conclusions may lead to label effects, which in turn worsen the mental health status of healthcare workers. In addition, we found that the detection rate of mental health problems in infected patients is higher in the COVID-19 pandemic than it was during the SARS outbreak [18]. For example, during SARS, the detection rate of anxiety symptoms was 35.7% (95% CI: 27.6–44.2), and that of depressed mood was 32.6% (95% CI: 24.7–40.9); in contrast, we found anxiety and depression rates of 63.9% (95% CI: 29.6–88.2) and 55.4% (95% CI: 32.8–76.0), respectively, in the COVID-19 context. During the outbreak of SARS in 2003, information dissemination was less developed than at present, and the public understanding of the virus was based on official information, which made the spread of rumors and concomitant psychological distress less likely. This shows that we should pay attention not only to the spread of the virus but also to the spread of false/fake information about the virus.

The second core finding of this study is that the detection rates of anxiety, depression, insomnia, and PTSD in other countries are higher than those in China. Existing study demonstrated the higher anxiety and depression symptoms in overseas Chinese lived in Italy than do of overseas Chinese lived in mainland China [19]. This may be because China was the first country to have an outbreak of the diseases and has taken a series of effective measures. Civil society organizations took responsibility for isolating residents in every community and helped solve practical life difficulties. At the individual level, home isolation, social distancing, and the wearing of personal protective equipment such as face masks were implemented to prevent community transmission nationwide. Due to the development of advanced technology, residents have had easy access to reliable information and medical guidance, which can reduce misinformation and the impact of rumors. The public was well educated on the seriousness of COVID-19 complied cooperatively with the national approach of hand washing, mask wearing, social distancing, and universal temperature monitoring. All citizens were keenly aware of their roles in preventing the virus from spreading. To strike a balance between epidemic control and normal social and economic operations, industrial activities have gradually resumed in phases and batches since February 8, 2020 [20]. The supply of daily necessities was kept stable in every stage of the outbreak to ensure the smooth operation of society. The WHO-China Joint Mission report said that China has rolled out perhaps the most ambitious, agile, and aggressive disease containment efforts in history [21]. By striking contrast, the number of confirmed cases outside China is quickly climbing following an exponential growth trend. The total number of COVID-19 cases outside China has reached 333,706,43, including 999,603 deaths as of September 29, 2020. Furthermore, we also conjecture that the reason why fewer pandemic impacts were seen in mainland China is that the well-established psychological rescue system strongly guards against the potential panic arising from the COVID-19 pandemic. Specifically, Chinese governmental intervention agencies provide professional psychological intervention services for patients with confirmed diseases or mental disorders, front-line medical staff, and other key groups in special places such as designated hospitals and isolated hospitals. In addition, public psychological rescue organizations offer free 24/7 on-call professional psychological advice to the public. Ultimately, massive open online courses were released to enrich the Chinese public’s understanding of the COVID-19 pandemic, which has significantly strengthened belief in the ability to control this disaster [22]. In addition, the comparative analysis of the results obtained with different measurement tools showed heterogeneity and poor consistency across the tools. Therefore, it is suggested that reliable measurement tools should be established in future research to avoid deviation in research results caused by measurement tools.

This study adjusted the prevalence of mental health problems reported in previous studies by analyzing more recent studies and thus provided a more accurate picture of the mental health status of the population. Previous studies have provided very timely and important evidence to prove that the COVID-19 pandemic is a threat to individual mental health. However, most of the surveys were performed in the early and peak periods and may overestimate the prevalence of these problems. Moreover, for the sake of timeliness in sharing research findings, low-quality articles were published in some journals. Therefore, this study also adopts the method of quality control evaluation to exclude articles with lower quality and obtain more accurate and unbiased conclusions. In general, the detection rate of mental health problems found in this study was lower than that in previous studies. There may be two reasons for this. First, stricter quality control was adopted in this study, making the analysis results more accurate and unbiased. Second, more new studies were included in this study; that is, the investigation time extended from the initial stage to the peak of the pandemic and then to the later stage of COVID-19 pandemic in the present study. Therefore, the results of this study may reflect that, with better control and understanding of the epidemic situation, people’s mental health status has improved, which is a good sign.

This study has several limitations. First, the sample sizes were not matched well, with the number of healthcare workers being smaller than the number of people from the general population. Second, the international sample was insufficient, and the research on Chinese people significantly exceeded than that on people from other countries. Third, the impact of specific epidemic status was not taken into account. In future studies, covariates can be added to the meta-analysis to control the epidemic situation of samples in different regions.

## Conclusion

In conclusion, our systematic review and meta-analysis provide a timely and comprehensive synthesis of existing evidence, confirming the presence of mental health problems in patients (including suspected patients) as well as insomnia and PTSD in medical staff. The findings help to quantify staff support in the context of a pandemic when stratified and customized interventions are needed to enhance resilience and reduce vulnerability. With the continuous emergence of new evidence, we can further update the meta-analysis and perform follow-ups to analyze the factors related to the epidemic situation to facilitate national-level planning, improve the hierarchical intervention of the mental health security system, and address similar public health events in the future.

## Supplementary information

SUPPLEMENTAL MATERIAL

## Data Availability

Study protocols and hypotheses were preregistered on the Open Science Framework (OSF) (https://osf.io/a5vmk/). Raw data, protocols, and analysis scripts are available openly at the OSF (https://osf.io/a5vmk/).
